# Biochemical assessment of selenium’s cardiovascular protective effects in a lipopolysaccharide-induced damage in rats: Focus on oxidative stress markers and IL-6

**DOI:** 10.34172/jcvtr.025.33496

**Published:** 2025-12-17

**Authors:** Farimah Beheshti, Mohammad Mahdi Sotoudeh, Mostafa Mansouri, Mohammad Mobin Mirimoghaddam, Yousef Baghcheghi, Mahmoud Hosseini

**Affiliations:** ^1^Neuroscience Research Center, Torbat Heydariyeh University of Medical Sciences, Torbat Heydariyeh, Iran; ^2^Applied Biomedical Research Center, Basic Sciences Research Institute, Mashhad University of Medical Sciences, Mashhad, Iran; ^3^Department of Physiology, School of Medicine, Torbat Heydariyeh University of Medical Sciences, Torbat Heydariyeh, Iran; ^4^Psychiatry and Behavioral Sciences Research Center, Mashhad University of Medical Sciences, Mashhad, Iran; ^5^Student Research Committee, Mashhad University of Medical Sciences, Mashhad, Iran; ^6^Bio Environmental Health Hazards Research Center, Jiroft University of Medical Sciences, Jiroft, Iran; ^7^Department of Physiology, School of Medicine, Mashhad University of Medical Sciences, Mashhad, Iran

**Keywords:** Selenium, Inflammation, Cardiovascular, Oxidative stress, Lipopolysaccharide

## Abstract

**Introduction:**

One of the main causes of illness and death in communities is cardiovascular disease (CVD). Inflammation and oxidative stress are key components in the pathophysiology of CVD. It has been demonstrated that selenium lowers inflammation and oxidative stress. The purpose of this study is to do biochemical assessment of selenium’s cardiovascular protective effects in a lipopolysaccharide (LPS)-Induced damage in rats.

**Methods:**

LPS+Selenium (100 µg/kg), LPS (1 mg/kg), LPS+Selenium (200 µg/kg), and Vehicle (instead of both selenium and LPS) were given to the four groups of rats. The rats were sacrificed after 14 days, and the serum, heart, and aorta were examined for the presence of malondialdehyde (MDA), thiol, catalase (CAT), and superoxide dismutase (SOD). Interleukin 6 (IL-6) was also assessed in the tissues of the heart and aorta as an indicator of inflammation.

**Results:**

LPS administration raised aortic and cardiac IL-6 levels (*P*<0.001). In the heart, aorta, and serum, it also raised MDA (*P*<0.001) and lowered thiol (*P*<0.001), CAT (*P*<0.01-*P*<0.001), and SOD (*P*<0.001). On the other hand, selenium therapy markedly raised thiol, CAT, and SOD levels (*P*<0.01-*P*<0.001) and lowered MDA levels (*P*<0.05-*P*<0.001). Furthermore, following selenium delivery, a decrease in the inflammatory marker IL-6 was noted (*P*<0.01-*P*<0.001).

**Conclusion:**

This study showed that selenium protected the heart, aorta, and serum from oxidative stress brought on by LPS. Additionally, it reduced aortic and cardiac inflammation. These results imply that selenium’s anti-inflammatory and antioxidant properties may help prevent or lower the morbidity and mortality of CVD.

## Introduction

 Cardiovascular disease (CVD) causes 17.3 million annual deaths, projected to rise to 23.6 million by 2030.^1,2^ It accounts for 46% of deaths in Iran and 39% in the U.S.^3,4^ Reducing risk factors is crucial to managing CVD’s health and economic impacts globally.^4,5^

 Oxidative stress and chronic inflammation are key, linked drivers of CVD, causing endothelial dysfunction, atherosclerosis, and heart failure.^6-9^ Oxidative stress arises from an imbalance between the production of free radicals, and the capacity of endogenous antioxidant defense systems to neutralize them. In the vasculature and myocardium, excessive free radicals directly damage cellular components (lipids, proteins, DNA), promote lipid peroxidation, inactivate nitric oxide (NO) leading to vasoconstriction, activate pro-inflammatory signaling pathways, and trigger cell death pathways.^7,10^

 Inflammation, closely tied to oxidative stress, involves immune activation and pro-inflammatory cytokines such as interleukin-1 beta (IL-1β), interleukin-6 (IL-6), and tumor necrosis factor-alpha (TNF-α). These mediators can cause ndothelial dysfunction, plaque formation, and heart damage.^9,11^

 Selenium (Se) is vital for health as a component of selenoproteins.^12,13^ Humans have over 25 selenoproteins, including glutathione peroxidases (GPx), thioredoxin reductases (TrxR), and selenoprotein P (SelP).^14,15^ GPx neutralizes peroxides, preventing oxidative damage; TrxR maintains redox balance; and SelP transports selenium and acts as an extracellular antioxidant.^16,17^ Consequently, Se status directly influences the activity of these crucial antioxidant enzymes. Selenium also regulates immunity and inflammation by inhibiting nuclear factor-kappa B (NF-κB)/ activator protein-1(AP-1), reducing inflammatory cytokines like IL-6 and TNF-α.^18-20^ Adequate selenium may lower cancer and CVD risks, but optimal and safe levels need further study.^21,22^

 Lipopolysaccharide (LPS), a key Gram-negative bacterial toxin, triggers inflammation and mimics sepsis or chronic inflammation in studies.^23,24^ By binding TLR4 on various cells, LPS triggers MyD88/TRIF pathways, leading to strong inflammatory responses dominated by TNF-α, IL-1β, and IL-6 production.^25,26^ The cytokine storm disrupts endothelial function, activates leukocytes and coagulation, and increases oxidative stress via nicotinamide adenine dinucleotide phosphate (NADPH) oxidases (NOX) and mitochondrial dysfunction.^27^ LPS models help study inflammatory/oxidative CVD mechanisms. Chronic low-dose LPS mimics’ sustained inflammation in metabolic syndrome and heart failure, reflecting endotoxemia’s role.^28,29^

 Se counteracts LPS-induced inflammation and oxidative stress in multiple organs by boosting selenoproteins (e.g., GPx) and reducing pro-inflammatory cytokines, as shown in diverse experimental models.^30-32^ Amini et al found selenium-L-methionine reduced lung inflammation and fibrosis in rats, while Wang et al showed Se protected mice from LPS-induced heart injury via the Sting pathway.^31,32^ Their work, utilizing an acute model (LPS injection 6 hours before sacrifice), provided valuable insights into rapid inflammatory and oxidative responses in the heart.

 This study addresses a key gap in Se research by investigating its cardioprotective effects in chronic inflammation-unlike prior acute models. Using a 14-day repeated LPS injection model (1 mg/kg/day) to mimic chronic CVD-linked inflammation, we evaluate dose-dependent Se supplementation (100/200 µg/kg) on oxidative stress (malondialdehyde (MDA), thiol, superoxide dismutase (SOD), and catalase (CAT)) and inflammation (IL-6) in heart, aorta, and serum. Unlike Wang et al’s acute Sting pathway focus, our work uniquely examines sustained Se effects in cardiovascular tissues, offering novel insights into chronic inflammatory and oxidative responses. Most prior research on Se and LPS has either focused on acute models or examined effects primarily in non-cardiovascular organs.^32-35^

 This study explores Se’s ability to mitigate chronic inflammation and oxidative stress in the cardiovascular system, addressing a key gap in existing research. Using a 14-day LPS-induced chronic inflammation model in rats, we investigated the dose-dependent effects of Se supplementation (100 µg/kg and 200 µg/kg) on oxidative and inflammatory markers in heart, aortic, and serum samples. Therefore, this study provides novel insights into the potential of Se to modulate chronic inflammatory and oxidative insults specifically targeting the cardiovascular system.

## Material and Methods

###  Animals

 In our study, we used 28 male Wistar rats with 220-240 g weight present in the Central Animal House of the Mashhad University of Medical Science. They were preserved in an environment with standard temperature (23-25°c) and a 12h light/dark cycle. Food and water were available as needed. Animals were randomly allocated to different treatment groups using a computer-generated randomization sequence. This method ensured that each animal had an equal chance of being assigned to any group, minimizing selection bias. We randomly divided the rats into four groups and they received the following treatments during 14 days^36^: (1) Vehicle, (2) LPS (1 mg/kg),^37,38^ (3) LPS + Selenium (100 µg/kg), and (4) LPS + Selenium (200 µg/kg). The treatments were done during 14 days. Eventually, we induced deep anesthesia with ketamine (100 mg/ kg) + xylazine (10 mg/ kg), the blood samples were collected. The heart and the aorta tissues of the rats were also removed. Oxidative stress in heart, aorta and serum was evaluated by measuring MDA, thiol, CAT, and SOD. Inflammation status was also estimated by measuring and IL-6. National Institutes of Health Guidelines for the Care and Use of Laboratory Animals were followed and the Animal Care and Use Committee of Mashhad University of Medical Sciences approved the experiments (IR.MUMS.MEDICAL.REC.1400.672).

###  Biochemical measurements

 A spectrophotometric method was used and MDA concentration in the serum, heart, and aorta were measured to show the lipid peroxidation level. MDA reacts with thiobarbituric acid (TBA), producing a red-colored complex. The peak absorbance of the complex is at 535 nm. Thus, we blended 1 mL of the homogenized tissues and 2 mL TBA/trichloroacetic acid (TCA)/hydrochloric acid mixture and then boiled it for 40 min. The final solution was centrifuged within 1000g for 10 min after cooling. Then, we measured the absorbance at 535 nm and calculated the MDA level.^39^

 In order to measure the total thiol groups, we used Ellman’s reagent, 2-nitrobenzoic acid (DTNB). The total thiol groups react with DTNB and produce the yellow-colored complex with a peak absorbance of 412 nm.^40,41^

 SOD activity was also evaluated with a spectrophotometric method according to Madesh et al study.^42^ In the absence of SOD, the tetrazolium molecule reduces by superoxide to red-colored formazan, which has an absorbance of 570 nm. The presence of SOD leads to the decomposition of the superoxide and decreases the production of red-colored formazan.

 We also assessed CAT activity by following the Aebi method.^43^ CAT breaks down hydrogen peroxide into water and oxygen. Homogenized tissues were combined with potassium phosphate and hydrogen peroxide. Sample absorbance was measured at 240 nm, and reduction of the absorbance was considered a high level of CAT activity.

 We evaluated IL-6 levels with an ELISA kit. Briefly, the samples (100 µL) were suspended to the wells. It was incubated at room temperature and then it was washed. The biotin-antibody was then added and the plate was incubated for an hour at 37°C. In the next step, an HRP-avidin was added to the wells, and the plate was incubated for another hour. The wells were washed 5 times. A TMB substrate was then added and the wells were incubated in the dark for 15 min. Finally, the stop solution was added and absorbance was read at 450 nm using a Biotech ELISA reader.

###  Statistical analysis

 All data were presented as mean ± standard error of the mean. ANOVA (one-way) test with Tukeys’ post hoc was used for data variance analysis. The differences were considered statistically significant when *P*< 0.05.

## Results

###  Normality of data

 In our study, we conducted normality tests using the Shapiro-Wilk test to assess the distribution of the data. The results indicated that the data were normally distributed, which supports the validity of our statistical analyses. This information has been included in the results section to enhance the clarity and robustness of our findings.

## Selenium attenuated inflammation in the heart

**Figure 1:**
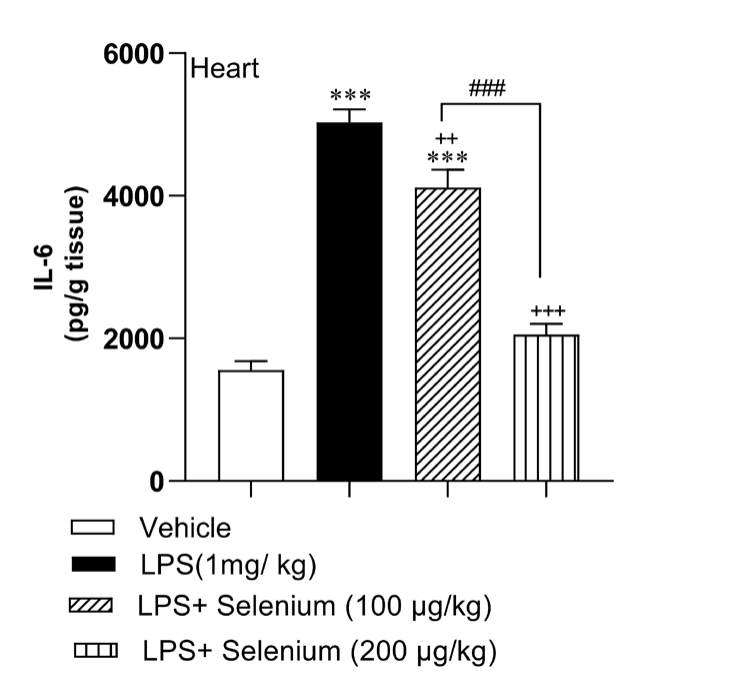
Effects of selenium on IL-6 levels in heart tissue after LPS challenge. ***P < 0.001 *vs *Vehicle group, ^+ +^*P* < 0.01 and ^+ + +^*P* < 0.001 *vs *LPS (1 mg/ kg) group,^###^*P* < 0.001* vs *LPS + Selenium (100 µg/ kg) group. Data are presented as mean ± SEM (n = 7)

 The results in heart tissue samples demonstrated that the mean level of IL-6 in the LPS-injected group was 5029 ± 182 pg/ g tissue and it was significantly higher than in the vehicle-treated group value (1558 ± 122 pg/ g tissue) (*P* < 0.001). Co-administration of 100 and 200 µg/kg selenium with LPS attenuated IL-6 concentration to 4118 ± 249 and 2056 ± 148 pg/ g tissue respectively, in the heart tissue of LPS + Selenium(100 µg/kg) and LPS + Selenium(200 µg/kg) groups compared to the LPS. The result showed that the higher dose 200 µg/kg of selenium was more effective than the lower dose 100 µg/kg in attenuating the IL-6 concentration in the heart tissue (*P* < 0.001). The IL-6 concentration in the heart tissue of the LPS + Selenium (200 µg/kg) group reached the control level but in the LPS + Selenium (100 µg/kg) group it was still higher than that in the vehicle-treated group (*P* < 0.001) (Figure 1).

###  Selenium attenuated oxidative stress in the heart 

**Figure 2:**
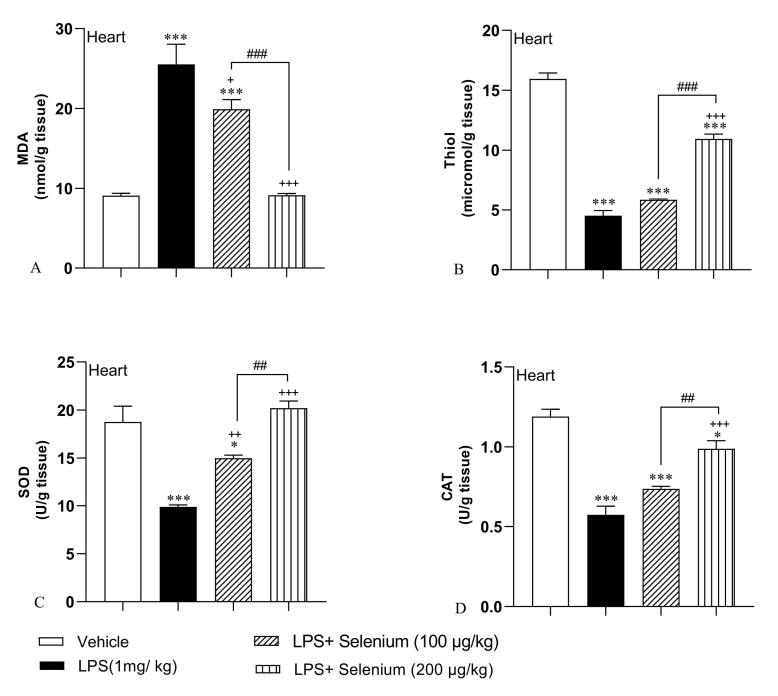
Effects of selenium on oxidative stress indicators in heart tissue after LPS challenge.*P < 0.05 and ***P < 0.001 *vs *Vehicle group, ^+^*P* < 0.05, ^+ +^*P* < 0.01, and ^+ + +^*P* < 0.001 *vs *LPS (1 mg/ kg) group,^##^*P* < 0.01 and ^###^*P* < 0.001* vs *LPS + Selenium (100 µg/ kg) group. Data are presented as mean ± SEM (n = 7)

 In addition, similar findings were shown in the MDA concentration of heart tissue. In the LPS group, MDA level was 25.52 ± 2.5 nmol/ g tissue and it was significantly higher than the control level (9.11 ± 0.27 nmol/ g tissue) in the vehicle group (*P* < 0.001). Selenium attenuated MDA levels in the heart tissue of LPS + Selenium (100 µg/kg) (19.9 ± 1.21 nmol/ g tissue) and LPS + Selenium (200 µg/kg) (9.13 ± 0.22 nmol/ g tissue) groups (*P* < 0.05 and *P* < 0.001 respectively). The higher dose of selenium was more effective than the lower dose and the MDA concentration in the heart of the LPS + Selenium (200 µg/kg) group reached the control level but it was lower than in the LPS + Selenium (100 µg/kg) group (*P* < 0.001). In the heart of the LPS + Selenium (100 µg/kg) group, MDA concentration was still higher than that in the Vehicle group (*P* < 0.001) (Figure 2A).

 It was also observed that heart tissue‘s thiol contents declined following LPS administration (4.52 ± 0.42 micromol/g tissue) compared to the control level (15.95 ± 0.48 micromol/ g tissue) in the Vehicle group (*P* < 0.001). Compared to the LPS group, treatment with the higher dose of selenium (200 µg/kg) improved thiol contents in the tissue (10.93 ± 0.40 micromol/ g tissue) significantly (*P* < 0.001) but the lower dose was not effective (5.84 ± 0.07 micromol/g tissue). Treatment with 200 µg/kg dose of selenium leads to a rising thiol level of more than 100 µg/kg (*P* < 0.001). In the heart tissue of both LPS + Selenium (100 µg/kg) and LPS + Selenium (200 µg/kg) groups, the thiol concentration was lower than in the Vehicle treated group (*P* < 0.001) (Figure 2B).

Figure 2C shows a significant decrease in SOD activity related to LPS administration (9.88 ± 0.18 U/ g tissue) compared to the activity in the Vehicle group (18.74 ± 1.63 U/ g tissue) (*P* < 0.001). In contrast, selenium at both doses significantly increased SOD activity in heart tissue of LPS + Selenium (100 µg/kg) (14.98 ± 0.31 U/ g tissue) and LPS + Selenium (200 µg/kg) (20.18 ± 0.73 U/ g tissue) groups (*P* < 0.01 and *P* < 0.001 respectively). The results indicated that 200 µg/kg Selenium administration leads to more SOD activity improvement than 100 µg/kg (*P* < 0.01). The SOD activity in the hearts of the rats treated with 200 µg/kg reached the level in the Vehicle group but it was lower than the control level in the heart of those animals treated with 100 µg/kg of selenium (*P* < 0.05).

 The findings also show that CAT enzyme activity was significantly lower in the heart of the LPS group (0.57 ± 0.05 U/ g tissue) than in the Vehicle group (1.18 ± 0.04 U/ g tissue) (*P* < 0.001). In addition, CAT activity significantly increased after treatment by 200 µg/kg dose of selenium (0.98 ± 0.05 U/ g tissue)in contrast with the LPS group (*P* < 0.001) but 100 µg/kg was not effective(0.73 ± 0.01 U/ g tissue). The CAT activity in the heart tissue in the rats treated with the high dose (200 µg/kg) of selenium was higher than those treated with the low (100 µg/kg) dose (*P* < 0.01). In the heart tissue of both LPS + Selenium (100 µg/kg) and PS + Selenium (200 µg/kg), the CAT activity was lower than the control level (*P* < 0.001 and *P* < 0.05 respectively)(Figure 2C).

###  Selenium attenuated inflammation in the aorta 

**Figure 3:**
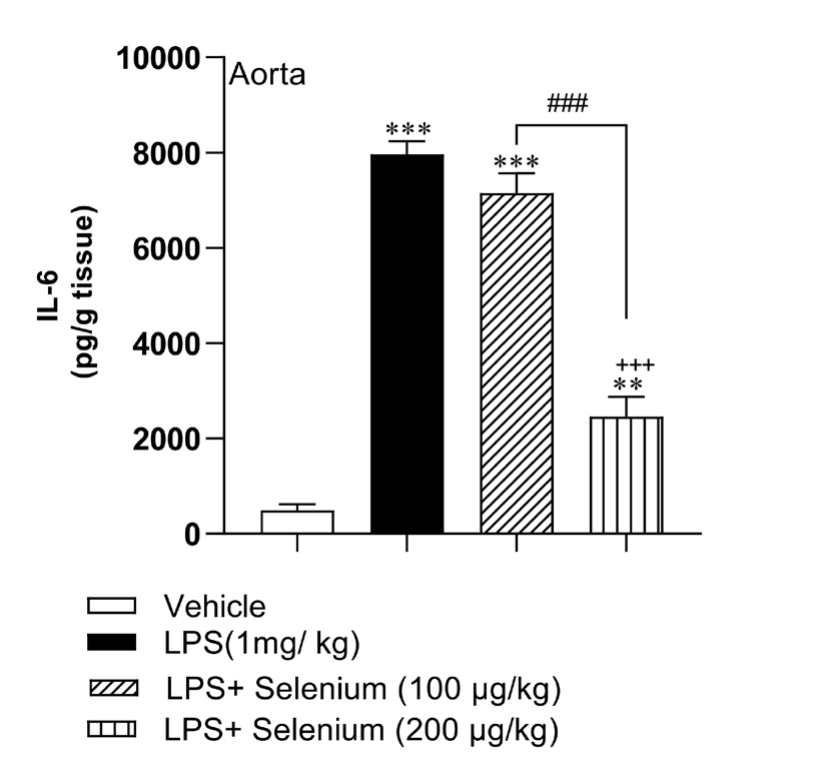
Effects of selenium on IL-6 levels in aorta tissue after LPS challenge. **P < 0.01 and ***P < 0.001 *vs *Vehicle group, ^+ + +^*P* < 0.001 *vs *LPS (1 mg/ kg) group,^###^*P* < 0.001* vs *LPS + Selenium (100 µg/ kg) group. Data are presented as mean ± SEM (n = 7)

 The results of IL-6 concentration in the aorta demonstrated that LPS injection induced an inflammation status in the aorta which was confirmed by an increased level of IL-6 concentration (7966 ± 273 pg/ g tissue) compared to the Vehicle group(490 ± 127 pg/ g tissue) (*P* < 0.001). The aortic tissue IL-6 in the LPS + Selenium (200 µg/kg) group (2459 ± 413 pg/ g tissue) was lower than the LPS and LPS + Selenium (100 µg/kg) (7152 ± 413 pg/ g tissue) (*P* < 0.001 for both) but there was no significant difference between LPS + Selenium (100 µg/kg) and LPS groups. The concentration of IL-6 in the aortic tissue of LPS + Selenium (100 µg/kg) and LPS + Selenium (200 µg/kg) groups was higher than that in the Vehicle group (*P* < 0.001 and *P* < 0.01 respectively) (Figure 3).

###  Selenium attenuated oxidative stress in the aorta

**Figure 4:**
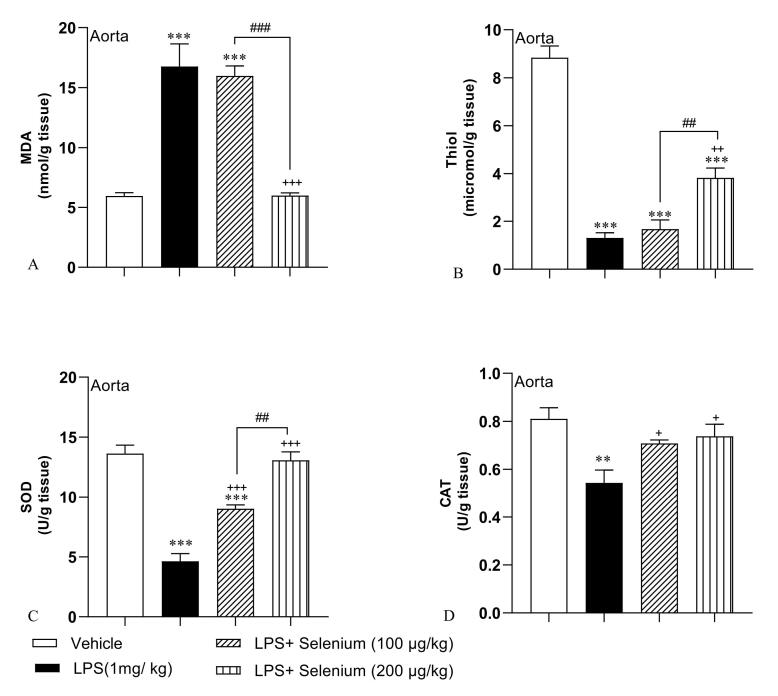
Effects of selenium on oxidative stress indicators in aorta tissue after LPS challenge.**P < 0.01 and ***P < 0.001 *vs *Vehicle group, ^+^*P* < 0.05, ^+ +^*P* < 0.01, and ^+ + +^*P* < 0.001 *vs *LPS (1 mg/ kg) group,^##^*P* < 0.01 and ^###^*P* < 0.001* vs *LPS + Selenium (100 µg/ kg) group. Data are presented as mean ± SEM (n = 7)

 According to our results in Figure 4, LPS injection was accompanied by oxidative stress in the aorta which was presented by an increased level of MDA (16.76 ± 1.88 nmol/ g tissue) and decreased levels of thiol (1.3 ± 0.21 micromol/ g tissue), SOD (4.62 ± 0.64 U/ g tissue), and CAT (0.54 ± 0.05 U/ g tissue) (*P* < 0.001, *P* < 0.001, and *P* < 0.01 respectively). The higher dose 200 µg/kg decreased MDA (5.99 ± 0.22 nmol/ g tissue) and increased thiol (3.82 ± 0.4 micromol/ g tissue) (*P* < 0.001 and *P* < 0.01 respectively) compared to the LPS-injected rats but the lower dose 100 µg/kg was not effective (MDA: 15.97 ± 0.82 nmol/ g tissue, thiol: 1.67 ± 0.38 micromol/ g tissue). Both 100 and 200 µg/kg selenium increased SOD (9 ± 0.31 and 13.07 ± 0.7 U/ g tissue respectively) (*P* < 0.001 for both doses) and CAT activity (0.70 ± 0.01and 0.73 ± 0.05 U/ g tissue respectively) (*P* < 0.05 for both doses). Aortic tissue MDA and SOD in the LPS + Selenium (200 µg/kg) group and CAT in both LPS + Selenium (100 µg/kg) and the LPS + Selenium (200 µg/kg) groups reached the control level but MDA in LPS + Selenium (100 µg/kg) group was higher and SOD was lower than that in the Vehicle group (*P* < 0.001 for both). In addition, aortic tissue thiol content in both LPS + Selenium (100 µg/kg) and LPS + Selenium (200 µg/kg) groups was lower than that in the Vehicle group (both *P* < 0.001). The MDA level in the aorta tissue of the LPS + Selenium (200 µg/kg) group was lower than that in the LPS + Selenium (100 µg/kg) (*P* < 0.001) but thiol and SOD were higher (*P* < 0.01 for both). There was no significant difference between the 2 doses of selenium when CAT activity was compared.

###  Selenium attenuated oxidative stress in the serum

**Figure 5:**
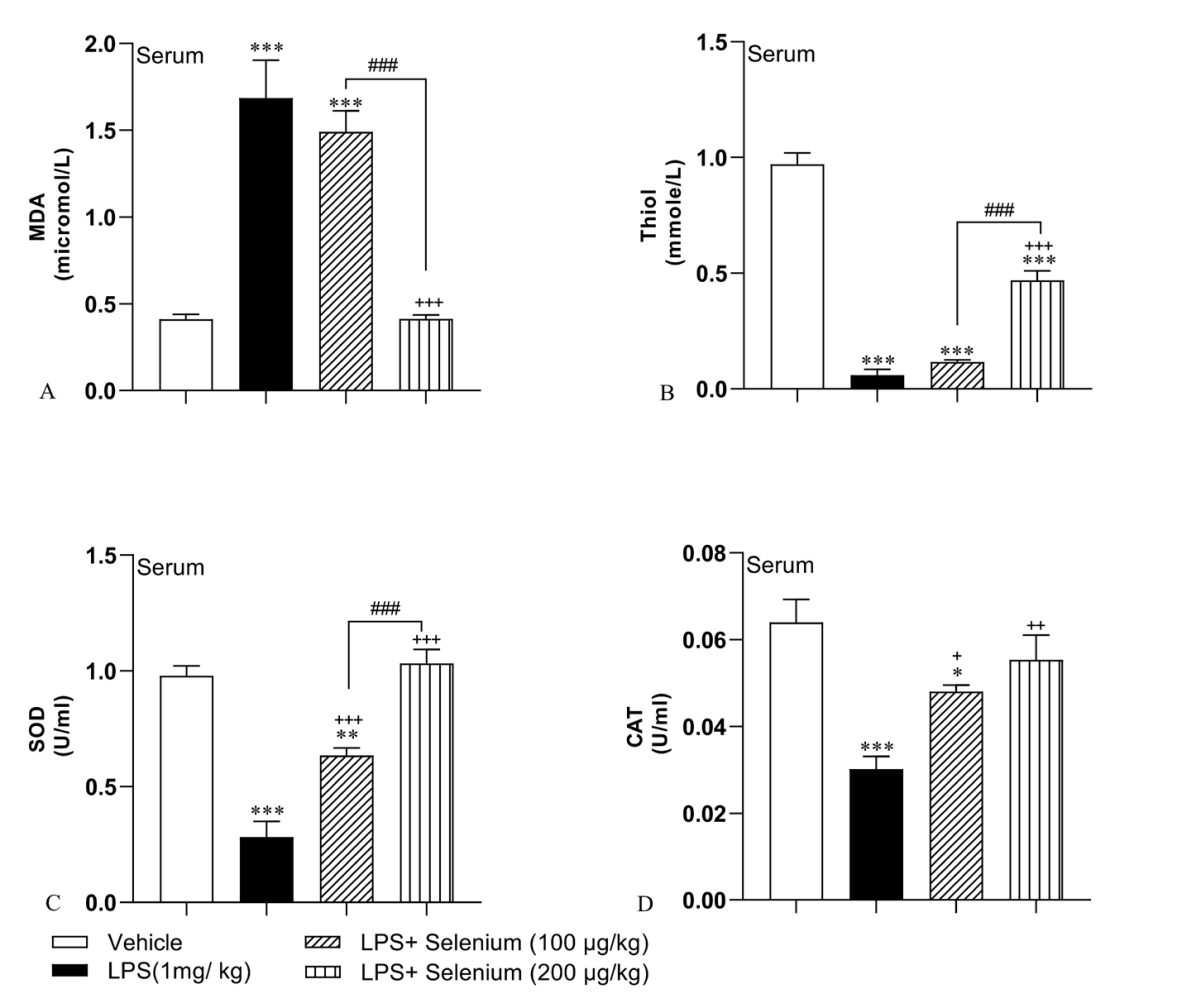
Effects of selenium on oxidative stress indicators in serum after LPS challenge.*P < 0.05, **P < 0.01, and ***P < 0.001 *vs *Vehicle group, ^+^*P* < 0.05, ^+ +^*P* < 0.01, and ^+ + +^*P* < 0.001 *vs *LPS (1 mg/ kg) group, ^###^*P* < 0.001* vs *LPS + Selenium (100 µg/ kg) group. Data are presented as mean ± SEM (n = 7)

 As it is shown in Figure 5, LPS injection was accompanied by an increase in MDA (1.68 ± 0.21 micromol/ L) and a decrease in thiol (0.05 ± 0.02 mmol/ L), SOD (0.28 ± 0.06 U/ L), and CAT (0.03 ± 0.002 U/ L), in the serum (*P* < 0.001 for all). Selenium administration in a high dose (200 µg/kg) could decrease MDA concentration (0.41 ± 0.02 micromol/ L) but increase thiol content (0.46 ± 0.04 mmol/ L)in the serum(*P* < 0.001 both) but 100 µg/kg was not effective (MDA: 1.49 ± 0.12 micromol/ L, thiol: 0.11 ± 0.007 mmol/ L). The biochemical results showed that both doses including 100 and 200 µg/kg improved SOD (0.63 ± 0.03 and 1.03 ± 0.06 U/ L respectively) (both *P* < 0.001) and CAT (0.04 ± 0.001 and 0.05 ± 0.005 U/ L respectively) (*P* < 0.05 and *P* < 0.01 respectively in the serum. The concentration of MDA in the serum samples of the LPS + Selenium (200 µg/kg) group was lower but thiol and SOD were higher than that in LPS + Selenium (100 µg/kg) group (*P* < 0.001 for all) but there was no significant difference between 2 doses of selenium in CAT activity in the serum. Serum MDA concentration of and serum SOD and CAT activities in the LPS + Selenium (200 µg/kg) group reached the control level but in the serum of LPS + Selenium (100 µg/kg) group MDA was higher (*P* < 0.001) and SOD and CAT were lower (*P* < 0.01 and *P* < 0.05 respectively) than that in the Vehicle group. In addition, serum thiol content in both LPS + Selenium (100 µg/kg) and LPS + Selenium (200 µg/kg) groups was lower than that in the Vehicle group (*P* < 0.001 for both).

## Discussion

 This investigation provides compelling evidence that Se supplementation confers significant protection against sustained, LPS-induced cardiovascular damage mimicking chronic inflammation, operating through the attenuation of oxidative stress and inflammatory pathways within cardiac and aortic tissues. Our findings robustly demonstrate that co-administration of Se alongside LPS for 14 days, particularly at the higher dose of 200 µg/kg, effectively countered the deleterious effects of prolonged inflammatory exposure. This was fonfirmed through a significant reversal of LPS-induced elevations in MDA and IL-6, alongside the restoration of depleted antioxidant reserves – thiol groups, SOD, and CAT activity – across serum, heart, and aortic compartments. While these observations align with the extensive literature documenting Se’s fundamental role as a cofactor for critical antioxidant enzymes like GPx and its broader immunomodulatory,^33,44-46^ the significance of this study lies in its detailed delineation of these protective effects specifically within the integrated cardiovascular system under a standardized model of chronic inflammatory insult induced by repeated LPS administration over two weeks.

 The novelty of our approach stems from several key design elements. Firstly, we concurrently evaluated the response of both cardiac muscle and aortic vasculature to prolonged LPS challenge, revealing parallel protection in these functionally distinct but interconnected cardiovascular tissues. While numerous studies have explored Se’s effects in isolated organs or in models of chronic disease,^32-35,45^ data directly comparing its efficacy in heart and aorta under systemic inflammation maintained by repeated LPS administration (1 mg/kg daily for 14 days) are particularly scarce. Our results demonstrate that Se not only protects the myocardium but also significantly mitigates oxidative and inflammatory damage within the aortic wall throughout this extended inflammatory period, suggesting a sustained benefit for vascular endothelial function and integrity during chronic inflammatory states, an area warranting dedicated functional studies. Secondly, the implementation of simultaneous Se and LPS co-administration for 14 days specifically models a scenario of ongoing chronic inflammation where therapeutic intervention is applied concurrently with the inflammatory trigger. This differentiates it significantly from studies using a single acute LPS bolus or therapeutic models applied only after chronic damage is established.^32,33,35,45^ This design directly tests Se’s ability to counteract persistent inflammatory stress relevant to conditions like chronic low-grade inflammation associated with atherosclerosis or heart failure^9^. Thirdly, the clear dose-dependent response observed throughout this chronic exposure, with the 200 µg/kg dose consistently outperforming 100 µg/kg across all measured parameters in all tissues, provides crucial practical insight for long-term intervention strategies. This dose-effect relationship underscores the importance of optimizing Se levels for maximal sustained protection within the cardiovascular compartment and suggests that the sensitivity of cardiovascular tissues to Se’s effects during chronic inflammation may differ from responses seen in acute models or other organs. This finding is critical for translational considerations targeting chronic cardiovascular diseases, as both deficiency and supra-nutritional Se levels can have complex biological consequences.

 The significant reduction in cardiac and aortic IL-6 levels by Se treatment provides strong evidence for its anti-inflammatory action within the cardiovascular system. IL-6 is a pivotal mediator of systemic inflammation, and it also exerts direct effects on cardiac function and vascular permeability. Its attenuation by Se aligns with known mechanisms where selenoproteins, particularly those in the TrxR and GPx families, modulate redox-sensitive signaling pathways like NF-κB. NF-κB activation is a central driver of pro-inflammatory cytokine production, including IL-6, in response to LPS.^19,20,47^ While our biochemical data confirm the downstream outcomes (reduced IL-6, restored antioxidant capacity), the absence of direct molecular pathway analysis (e.g., NF-κB p65 phosphorylation, nuclear factor erythroid 2-related factor 2 (Nrf2) nuclear translocation) represents a limitation acknowledged. Future mechanistic studies should explicitly link Se supplementation to the inhibition of these key inflammatory and oxidative stress master regulators within cardiovascular tissues. Furthermore, while the concordant elevation of IL-6 and oxidative stress markers (MDA, depleted antioxidants) across serum, heart, and aorta robustly supports the induction of a systemic inflammatory state impacting the cardiovascular system, the inclusion of additional inflammatory mediators (e.g., TNF-α, IL-1β) and histological assessment of tissue inflammation (e.g., leukocyte infiltration, cardiomyocyte/vascular damage) would have provided a more comprehensive validation of the model and a deeper understanding of Se’s tissue-level protective effects beyond circulating or homogenate markers. Histopathology, in particular, could have visually corroborated the biochemical evidence of protection by revealing reduced tissue damage.

 The broader context of our findings resonates with studies demonstrating Se’s protective effects against LPS-induced injury in other organs.^32,35,48,49^ The observed reduction in MDA and enhancement of SOD/CAT activity mirror results seen in hepatic, renal, and neuronal damage models,^44,50-53^ confirming the fundamental antioxidant properties of Se. Similarly, the suppression of IL-6, TNF-α and NF-κB signaling pathways aligns with Se’s documented ability to dampen pro-inflammatory cytokine cascades.^34,45,48,54^ However, the unique contribution here is the quantification of these effects simultaneously within the heart and aorta, highlighting a coordinated cardiovascular protection. This specificity is important, as tissue responses to both LPS and antioxidants can vary significantly. The superior efficacy of the 200 µg/kg dose compared to some studies where lower doses were effective might reflect the severity of the single high-dose LPS challenge used here or inherent differences in the baseline Se status or selenoprotein expression profiles within rat cardiovascular tissues compared to other models. The use of sodium selenite, while common, represents one specific form; exploration of organic Se compounds (e.g., selenomethionine) or specific selenoprotein mimetics in future work could yield different dose-response relationships or tissue-specific effects.

 Several limitations inherent in the study design must be considered when interpreting these results. Primarily, the chronic LPS injection model, while valuable for studying initial inflammatory and oxidative responses, does not fully recapitulate the complex, prolonged pathophysiology of chronic CVD like atherosclerosis or heart failure. Extrapolating our findings directly to long-term CVD morbidity and mortality reduction in humans requires caution. Secondly, the focus on biochemical markers, despite providing clear quantitative evidence, leaves gaps in understanding the underlying molecular mechanisms and the ultimate impact on tissue structure and function. The absence of data on apoptosis, specific signaling pathways (NF-κB, Nrf2, mitogen-activated protein kinase (MAPKs)), and histopathological correlation limits the depth of mechanistic insight. Thirdly, reliance primarily on IL-6 as the indicator of inflammation, although showing a consistent response across compartments, could be strengthened by measuring a broader cytokine/chemokine panel and adhesion molecules, particularly within the vascular endothelium. Finally, the single high-dose LPS model differs from chronic low-grade inflammation models or repeated endotoxin exposure paradigms relevant to some CVD contexts.

 Despite these limitations, the study possesses significant strengths: a randomized, blinded design; the systematic evaluation of key oxidative and inflammatory markers in serum, heart, and aorta within a single experimental framework; the demonstration of a robust, dose-dependent protective effect specifically within the cardiovascular system. The protocol specifically highlights the potential of prophylactic/preemptive Se administration in settings of anticipated or acute systemic inflammation.

## Conclusion

 In conclusion, this work solidifies the concept that Se, particularly at an optimized dose of 200 µg/kg, provides significant cardioprotection in the face of systemic inflammatory stress induced by LPS. It achieves this by effectively combating oxidative damage and suppressing key inflammatory mediators like IL-6 within both the myocardium and the aortic wall. While confirming the established antioxidant and anti-inflammatory properties of Se, the study advances the field by detailing a coordinated, dose-responsive protective effect specifically across the cardiovascular compartment under acute duress. These findings underscore the potential of Se supplementation as a strategic intervention to mitigate acute cardiovascular complications arising from severe systemic inflammation, such as in sepsis or during inflammatory episodes in susceptible individuals. Future research must prioritize elucidating the precise molecular mechanisms (e.g., Nrf2/ antioxidant response element (ARE) activation, NF-κB inhibition) within cardiovascular cells, incorporate functional assessments (e.g., echocardiography, vascular reactivity, endothelial function), perform detailed histopathological analysis, explore different Se compounds and dosing regimens, and evaluate efficacy in chronic inflammation models relevant to prevalent cardiovascular diseases to fully unlock its therapeutic potential.

## Competing Interests

 The authors have no conflict of interest to declare.

## Ethical Approval

 The Animal Care and Use Committee of Mashhad University of Medical Sciences approved the experiments (IR.MUMS.MEDICAL.REC.1400.672).
